# Book Reviews

**Published:** 1802-02

**Authors:** 


### CRITICAL RETROSPECT
OF
### MEDICAL AND PHYSICAL LITERATURE.

[ FOREIGN AND DOMESTIC. ]

---

### *Observations on the Cancerous Breast, by Dr. Adams.*

[Continued from our last, pp. 82—89.]

The peculiar nature of the *fat* of a carcinomatous breast is likewise very forcibly contrasted with the healthy secretion.

" You admit the propriety of my remark, that the fungus divides the fat into distinct portions; but you object to my addition, that the fat thus enclosed should be free from *common* cellular substance. Thus then we agree that there is a fungus, or if you prefer the term, a scirrhus, which divides different portions of fat from each other.

" Before I explain the peculiarity of this enclosed fat, let me in my turn beg you to account for the great quantity of fat you find in a truly carcinomatous breast. Compare the size of the two breasts in the same subject, and examine how much of the increased size in the diseased one is made up of fat. Is it probable that such an increased quantity should be a healthy secretion, or that it should even be deposited by any process similar to what takes place in a state of health? I admit with you that it has the same appearance, and also that it has a cellular apparatus. But this appearance is only the same when the amputated part has been kept long enough for the fat to consolidate, and the cellular apparatus is altogether different. Instead of the apparently irregular net-work, which forms the common adipose cellular membrane, and which is so entirely intermixed with fat, that without cutting into small pieces and dissolving by heat, it is impossible to separate fat from membrane, the cells of true carcinoma are so many small and firm capsules, each containing its distinct portions of fat, without connection of each other but by lateral adhesion."

Having raised his theory of the hydatid-nature of carcinoma on a foundation which many of our readers will probably consider of very doubtful solidity, Dr. Adams proceeds to a superstructure still more bold and original, which is the animalcular nature of *steatoma*. The author indeed is aware of the objection which instantly suggests itself in the total want of *motion* or *muscular contraction* in steatomatous tumours; which property he had endeavoured to exhibit in the papillary appearance assumed by the cells of carcinoma when cut transversely, after all connection with the body had been removed

 by by amputation. He obferves with perfect propriety, that as muf-cular contraction is confidered by Dr. Baillie as one of the proper-ties of life, and the ftrongeft proof of its prefence, and as carci-noma poffeffes this property but fteatoma does not, the proofs of the vitality of the former are independent of any analogy with the latter; and therefore, as they are not fupported by fuch ana-logy in fome particulars, they do not fail from the want of it in others.

This obfervation however only applies to the queftion of the vi-tality of carcinoma, and the objection which will be directly urged againft the feparate exiftence of fteatoma, from its want of muf-cular contraction, remains in its full force. This objection our readers will perhaps think, is hardly obviated by the following re-mark: "Though no contraction can be perceived in the tunics of "moft other encyfted tumours," (than carcinoma) "yet this may "arife from the contents of fome being too folid, and others too "fluid, to form the papillary appearance I have defcribed in car-"cinoma." In a note, however, the author obferves, that in the watery encyfted tumour (or the lymphatic hydatid in a folid part as he would term it) on being punctured before its death, the contain-ed fluid, though turbid, efcapes with confiderable force like water from a compreffed bladder; which he is inclined to impute to muf-cular contraction.

The arguments for the feparate vitality of fteatoma and tumours of a fimilar nature, the author gives in the following terms, "Thus, without further preface, I am free to acknowledge that not only fteatoma, but atheroma and meliceris, as they have been called fince the days of the Greek phyficians, that is, all encyfted tumours, whofe cyft and contents have no communicating branches with the furrounding blood veffels, appear to me animalcular, or at leaft to have the fame œconomy as has been admitted in hydatis lympha-tica.

That this is the cafe I conceive:

Firft, Becaufe they are all found in the fame parts of the body, and often in the fame individual tumour.

"Secondly, Becaufe they are all free from any communicating branches in the furrounding blood-veffels.

"Thirdly, Becaufe they all appear to have a power of growth, after which they die, without otherwife affecting the body in which they exifted, but by their local ftimulus.

Fourthly, Becaufe the cyft containing either of them is inca-pable of fuppuration, and fubject to none of thofe laws, by which capfules formed to prevent the diffufion of matter in abfcefs, or fup-puration, or original tunics when preternaturally diftended with fluid, are governed.

"Fifthly, Becaufe a fimilar mode of multiplication may be traced in each."

The firft pofition he illuftrates by the examples of the breaft, uterus, and tefticle; and the abdomen, in which fteatoma is often found found in the fame cyft with the lymphatic hydatid, from the authority of Haller and others.

The fecond argument is univerfally admitted without difpute. The author likewife notices the difference betwen the cyft of a watery encyfted tumour and the capfule of a common abfcefs. The latter, Mr. Hunter has fhewn to be formed by an effufion of coagulable lymph which confines the pus within the cyft, and alfo plugs up the extremities of any contiguous blood veffels, fo as to prevent hæmorrhage. On the other hand, the tunic of encyfted tumours is compofed of regular concentric ftrata (of which Dr. A. has feparated not lefs than ten) and the blood veffels are not obliterated fuddenly by coagulated lymph, but ramify with extreme minutenefs over the coats of the cyft, but never in any degree enter its contents, whether folid or fluid.

The third pofition, Dr. A. attempts to eftablifh by fuppofing that the uncertain progrefs and often ftationary condition of fteatoma may be fuppofed to be the *torpidity* of the hydatid, fimilar to the ftate of the egg before incubation; and that the fuppuration of meliceris and the gangrene in fteatoma are the death of the hydatid, which then acts as an extraneous fubftance within the living body, and thus becomes difcharged.

The neceffity of diffecting away every part of the fac of encyfted tumours in the operation for their removal, and the impoffibility of making it the bafis for granulation and fuppuration, prove a very marked difference between thefe tumours and common abcefs, and fufficiently illuftrate the author's fourth argument.

The fifth pofition Dr. A. endeavours to prove by the known progrefs of feveral cafes of fteatoma, in which the difeafe increafed by cyfts or in clufters. The author concludes this laboured and ingenious illuftration by pointing out the peculiar points of difference between fteatoma in its various forms and carcinoma; the principal of which is, the property which the latter poffeffes of producing fungus, as has been already mentioned.

The concluding letter in this volume is addreffed to Dr. Stokes, and contains, along with a recapitulation of the author's peculiar opinions, feveral practical remarks on the treatment of cancer. Dr. A. denies that the corrugated rednefs (from which the term *cancer* was fuppofed to derive its origin) arifes from varicous veins, but thinks that the appearance is to be attributed only to the thinnefs of the integuments previous to ulceration. The author's opinion on the origin of the term cancer is at leaft as plaufible as the other. It is as follows:

"As to the term cancer, it appears to me that the ancients had a much better reafon for the ufe of it; I mean its property of proceeding *backwards*, or contrary to the progrefs of abfceffes, which is towards the furface. Whilft the fungus is extending to the fkin, the carcinomatous hydatids are multiplying internally, and feem to avoid the fkin as much as poffible. When ulceration has begun, it is not, as in common abfcefs, becaufe matter has approached the furface with a previous elongation and, as we call it, pointing of the fkin;

ſkin; for the fluid, which conſiſts only of hydatids altered in their form by the loſs of life, makes no progreſs towards the ſurface, but remains till the gradual ſloughing and ulceration of the fungus expoſe the cavity in which they are contained. When by theſe means the fragments of the cyſts and turbid fluid into which the hydatids are converted by death eſcape, the ſides of the cavity do not collapſe like that of common abſceſs, but expoſe a ghaſtly cavern, from which, inſtead of pus, a watery diſcharge is ſecreted, attended with a very peculiar ſmell. Round the mouth of this cavern, fungus ſometimes grows; and inſtead of any attempt to unite, the ſkin curls in a contrary direction, which increaſes the depth of the cavity. This continues as long as any fragments of hydatid-tunics retain their life. As they die, ulceration takes place to detach them, like other extraneous bodies; and this ulceration being unattended with fungus, renders the cavity for a time ſtill deeper. After all the fragments of tunics are detached, the wound will perhaps continue ſtationary or heal, or more commonly another ſet of hydatids will be ſo far advanced as to produce a freſh ſloughing or ulceration.

"If this progreſs of the diſeaſe from the ſurface inwards was ſufficient to admit the alluſion to the ſuppoſed motion of a crab, the more rapid caſes might with ſtill more juſtice; for in theſe the patient will ſometimes be deſtroyed before the ſkin is broken."

Dr. A. agrees with his correſpondent, that in a warm climate this dreadful malady is leſs frequent and leſs ſevere than in cold.

The ſucceeding obſervations relate to the important queſtions, Whether the operation ſhould be performed at all? and, Whether any other mode of treatment will produce a cure? The author adduces the oppoſite teſtimony of two highly reſpectable authorities, the late Dr. Alex. Monro, and Mr. Hill. The former obſerves, that of ſixty cancers, at the extirpation of which he had been preſent, only four patients remained free from the diſeaſe for three years (a very alarming prognoſtic for the operator!) Mr. Hill on the other hand ſays, that he has extirpated no leſs than eighty-eight genuine cancers, eighty-four of which were ulcerated, and all excepting two recovered of the operation. Mr. Hill's caſes however were probably, he thinks, chiefly of the lip, which are leſs formidable than in moſt other parts. Dr. A. alſo notices the valuable ſtatement given by Mr. Fearon; and he adds the following remark, in which we believe he will be very feelingly joined by a great number of practitioners.

"If I were to give the reſult of my own obſervation, I know not how to account for it, but where every thing has appeared to ſucceed, the patients have in many inſtances died within a year or two after the operation. Where they have lived longer, the diſeaſe has ſometimes re-appeared in a few months; ſometimes at a period ſo remote, that all apprehenſions concerning it were at an end. It is however much more ſatisfactory to give the reſult of other mens' practice than our own, eſpecially when their caſes are related without any bias towards our own opinion."

The

The following injunctions for the performance of the operation merit attention.

"The firft ftep appears to me too often neglected, which fhould be to compare the difeafed with the found breaft; and if we find a greater fulnefs in the former towards the neck and axilla, whether attended with ftony hardnefs or not, we may depend on it that the difeafe extends beyond the indurated part. If a hardnefs in the axilla is attended with this fulnefs in the parts above-mentioned, we have every reafon to believe that the multiplication of hydatids extends regularly from one perceptible induration to the other, though no fungus can be felt except in thofe parts where the hydatids, from the greater rapidity of their growth and multiplication, or from their approaching nearer the fkin, have ftimulated the parts to the production of this fungus. In this cafe, to remove all the difeafe would be to remove the whole breaft and neighbouring cellular fubftance, extending fuperficially from the fternum to the axilla, and in depth to the pectoral mufcle under the clavicle and ftill more inacceffible parts.

"If at the fame time the fkin fhould be fo much difeafed, that it would not be eafy to leave enough to cover fuch a furface, nothing could excufe fuch an operation but the importunity of the patient, or a confidence in the operator, that he has courage and perfeverance equal to fuch an undertaking.

If the lump feems circumfcribed and the axilla clear, the next enquiry is, whether every other part of the breaft appears fimilar to the found one. In this examination fome latitude muft be admitted, the left breaft being ufually fomewhat larger than the right. But this muft be referred to the recollection of the patient at a period before the difeafe could have been fufpected. We muft alfo make allowance for any occafional exacerbations of inflammation, which extending much beyond the tumour by producing an increafe in the fize and number of veffels, will for a time enlarge the whole breaft."

After the mafs of the carcinomatous tumour is diffected out, it is ufual for the operator to determine whether the whole difeafe is removed, by feeling if any hardened lumps ftill remain.

To this Dr. A. obferves, that "Nothing can be more fallacious than fuch an attempt. The furface is often fo much covered with blood as to render an accurate infpection impracticable, and carcinomatous hydatids may exift without a fungus, and confequently without a perceptible induration. Our attention therefore fhould be directed to the cut furface of the amputated part. This we can examine in the moft favourable points of view without incommoding our patient, or without being interrupted by any bleeding veffels. If we find on its furface either an appearance of fungus or of that greenifh yellow fat which I call the carcinomatus hydatids, or any thing different from the natural healthy ftate, we muft apply to the correfponding furface of what is left, and renew our operation till what we cut off has a natural healthy appearance."

Concerning the ufe of the arfenical cauftic, the author is decidedly edly of opinion that the few cafes in which this practice has fucceeded, have been thofe of fcrophulous or fteatomatous tumours, in which the whole fac with its contents has floughed out, and left a clean granulating furface to fill the cavity thus left. He obferves however, and we think very juftly, that true carcinoma will be dreadfully exafperated by the cauftic mode of extirpation; and that the want of integuments whereby to cover the wound and heal it by the firft intention, is a moft ferious objection in a wound fo difpofed to the formation of fungus.

A variety of obfervations on the ufe of internal remedies conclude this letter, and with it the volume.

We have thus given our readers a very detailed account of this ingenious and very original treatife. We cannot entirely fay that the author has made out, in a fatisfactory manner, the juftnefs of his fingular hypothefis; but the work fhews fo much acute reafoning and accurate obfervation, that it will enfure to its readers entertainment and profit, if it fhould not enforce conviction.

---

*A Treatife on the Cow-pox; containing the Hiftory of Vaccine Innoculation, and an account of the various publications which have appeared on that fubject, in Great Britain, and other parts of the world. By* John Ring, Member of the Royal College of Surgeons in London. Part I. 8vo. pp. 496. London, 1801. Carpenters.

The author explains his reafons for publifhing this elaborate work in *parts* in his Preface. "The vaft number of remarks publifhed on Vaccine Innoculation, both at home and abroad, having fwollen this Treatife far beyond the bulk at firft intended, it was deemed expedient to divide it into two parts. The continual occurrence of new facts, and publication of new treatifes on the fubject, rendered it impracticable to preferve a ftrict and methodical arrangement. The intention of the author was, to collect and combine the fubftance of all that has hitherto been afcertained on this interefting fubject; and rather to incur the cenfure of prolixity, than to deferve the charge of omitting any thing of importance, on an occafion where the welfare and happinefs of the whole human race are fo immediately concerned.

"In fome meafure to fupply the want of fyftematic order, a copious Index will be fubjoined to the fecond part. Two plates will accompany that part, which will unavoidably caufe an addition to the price. The difficulty of procuring accurate reprefentations of the Vaccine Puftules has delayed the publication of this work; and, it is hoped, the reafon, when underftood, will plead a fufficient apology for that delay. It was the author's wifh, to have given one plate with each volume; but he was unwilling longer to defer, what, he fincerely hopes, may prove ufeful."

Mr. Ring then, with his well known ability for the tafk, purfues the hiftory of this very interefting fubject from the firft mention of it in London down to the prefent time. He does not, however, think it neceffary to follow chronological order on all occafions; but, but, on the contrary, he brings all the material evidence which may tend to illuftrate any important fubject, as far as poffible, into one point of view, though developed at different times. For inftance, the information refpecting the origin of the virus, received only a few months ago, will be found under that head, at the beginning of the prefent volume, to page 32. There can be no doubt that every Practitioner, who thinks it his duty to acquire the moft ample and correct information on Vaccination, will perufe Mr. Ring's elegant and comprehenfive work. Our readers will not expect that we fhould give extracts from Mr. R's Treatife, becaufe we have devoted fo large a portion of the Journal to the elucidation and confirmation of this fubject from its commencement. The zeal and perfeverance with which Mr. R. has traced and expofed the falfhoods and misreprefentations, fabricated by ignorant, envious, and malignant practitioners, merit the applaufe of his cotemporaries, and will fecure that of pofterity.

---

*Osservazioni Pratiche sull'uso del Vajuolo Vaccino, come Preservativo del Vajuolo Umano*; i. e. *Practical Observations on the Use of the Cow-pox, as a Preservative against the Small-pox.* By LEWIS SACCO, M. D. 8vo. pp. 216. Milan.

*( With Plates. )*

SOME extracts from this publication cannot fail of being interefting to the public, as they fhew in what light the difcovery of the Cow-pox is confidered among foreign nations. The author is a medical man of great eminence in Italy, and it is probable, from his great fuccefs, that his fentiments will be fpeedily adopted over all that country. In his Introduction, he thus fpeaks of the Cow-pox, and the great advantages refulting to mankind from its difcovery.

" Among the very great number of experiments which our age boafts, one of the moft important, and one which will make an epoch in the annals of medicine and of humanity, is the Inoculation of the Cow-pox, by means of which man is preferved from the infection of the natural Small-pox, and its dreadful confequences. Still indeed we are unable to trace the connection that exifts between the pus of the ordinary Small-pox, and that which is extracted from certain puftules which appear under the udders of cows, and which on that account are called the Cow-pox; but it has been happily demonftrated, that the latter being introduced into the human fyftem, after a fhort period of fymptoms by no means fevere, becomes a complete prefervative againft the Small-pox, which, though fometimes a flight difeafe, frequently deforms the perfon, inflicts irreparable wounds on the moft tender parts of the frame, and often ends in its deftruction. The difcovery of inoculation for the Small-pox furnifhed the means of preventing, in a great meafure, thefe difafters; but this practice was very flowly adopted, and we in Italy particularly fuffer ourfelves ftill to be expofed to the ravages of the natural Small-pox. It would be a long detail, were I to recount all the teftimonies of celebrated writers concerning the horrible ravages of this difeafe. According to the moft moderate calculation, ten out of every hundred attacked by the the natural Small-pox, fall victims to it; and if we estimate by this computation the number who are cut off by its ravages, during the course of twenty-five years, Europe in that period suffers from this cause the loss of fifteen millions of its inhabitants. In America, where the natural Small-pox is of a still more fatal nature, it carries off twenty, thirty, and even forty in every hundred."

After having produced many other striking facts, which are already well-known in this country, to shew the disastrous effects of the natural Small-pox, and the benefits arising from inoculation; the author proceeds to the discovery of Dr. Jenner, justly extols it as a benefit conferred on the human race, and proves, from the united results of numberless experiments, that it completely prevents any attack of the natural Small-pox, and is infinitely more safe and easy in its operation than the mildest inoculated Small-pox.

He then pays the following just tribute to the unwearied industry and perseverance of Dr. Jenner, lamenting at the same time the slow and "unwilling gratitude of base mankind."

"But this discovery, so fortunate for the human race, shared the fate of other grand and useful innovations, by encountering much opposition at its first outset. The basest envy let loose all its virulence against the discoverer on his appearance in London; but its attacks only made him redouble his diligence to bring his discovery to perfection. For a moment he retired from his enemies, to confound them on his return with the victorious arms of multiplied observations, and the most decisive experiments. At a distance from the clamours of a populous city, in the retirements of Gloucester, where the Cow-pox is almost endemic, Jenner had an opportunity of continuing his observations in the fullest tranquillity; and after a very great number of experiments, he resolved no longer to doubt of the immense advantages to be derived from the results he had obtained. At the same time he wrote down the whole series of his observations; and placed his ideas in such a clear and strong light, that he excited in every one a desire to examine their justness, by following the steps of the author in his investigations. The most eminent medical men in England began to turn their attention towards the Cow-pox. And as it is the fate of all the most useful discoveries, to meet with opposition and obstacles, which, by exciting the general curiosity, fix the public attention upon them, Jenner reaped this advantage from the virulence of his enemies, and after many struggles between prejudice and good sense, between various passions and the direct guidance of reason, truth finally triumphed, and the most celebrated medical men of England, convinced by their own experience and the observations of others, declared themselves advocates for the Cow-pox.

"The inoculation of the Cow-pox was carried over in a short time to our continent, and was most readily adopted in France, where that enlightened government, with the assistance of the National Institute, made the experiments be repeated, and afterwards multiplied multiplied with a like happy fuccefs through the departments of the Republic. At Ginevre particularly, the chief town of the department of Lemagne, many learned and enlightened men fet the example of following with a folicitous attention the fteps of the Englifh difcoverer. Here a great variety of experiments were made on the Cow-pox, in which the illuftrious profeffor and phyfician Odier, as well as Tourlet and Pictet particularly diftinguifhed themfelves; and publifhed on that fubject moft inftructive memoirs, which have ferved as a guide to medical men in many other countries, and prompted them to endeavour likewife to deferve well of their country and of the human race. About the fame time, the Cow-pox was introduced into Germany, and with fuch fuccefs as to be generally fubftituted to inoculation for the Small-pox, as it had been done in England and France. Notwithftanding the evidence of thefe facts, and of this common confent of the greateft nations in adopting the Cow-pox, there were not wanting, both in France and Germany, opponents of the fame clafs with thofe that Jenner had to encounter in England. Some of them, before knowing the refults of experiment, began to oppofe it, and to decry the inoculation of the Cow-pox as a dangerous practice, that introduced into the human fyftem a morbid matter taken from brutes. But this fort of oppofition was foon forced to give way to the immenfe number of experiments, not one of which was clouded by an unfavourable iffue; and the enemies of the Cow-pox were in a fhort time reduced to a filence fortunate for humanity.

"In Spain alfo, which vies with other cultivated nations in ufeful ftudies, and in application to thofe things that regard the profperity of fociety, the Cow-pox began to be inoculated; and the firft attempts having fatisfied the moft fanguine hopes of fuccefs, will no doubt foon render the practice general in that vaft kingdom and its populous colonies. I have been certified of thefe particulars by Seignor de Condado, his Catholic Majefty's Chargé d'Affaires with the Cifalpine Republic, a man equally refpectable for his learning, his philanthropy, and the diftinguifhed rank he holds as the reprefentative of his court. He likewife communicated to me the following article, which appeared in the Madrid Gazette, January 6, 1801, and which is to be confidered as authentic. "Dr. Don Francefco Piguillem, a phyfician of Puigerda, wifhing to find the truth of thofe prodigies attributed to the Cow-pox by the moft celebrated French and Englifh phyficians, caufed a portion of the virus or vaccine matter to be fent him from Paris; and with it he inoculated four boys, the 3d of laft December. Although the operation was performed in the coldeft and moft fevere days of this winter, the eruption took place, and followed its courfe with the greateft regularity, without the boys lofing their chearfulnefs even a fingle day, or fuffering the fmalleft uneafinefs. On the 15th of December, which was the 12th of the inoculation, the fame profeffor vaccinated fome other boys with matter taken off the firft; and this fecond operation was performed in the prefence of the governor of the city and other perfons of

 diftinction.

diſtinction. The benign nature of the Cow-pox, the regular courſe it obſerves, and the reſult, which was entirely conformable to the deſcriptions of the Engliſh and French phyſicians, makes Dr. Piguillem hope that this invention will in a few years entirely baniſh the Small-pox."

"But ſince the inoculation of the Small-pox has not met among us with the reception it deſerved, and ſince by this means the courſe is left free to the deſtructive influence of the natural variola, every good Italian ought to hope, that in the preſent happy epoch, he will ſee adopted the Vaccine Inoculation, which poſſeſſes ſo many and ſo remarkable advantages over the former inoculation. It is deſigned as the ſafe-guard of the human race againſt the natural Small-pox; and I have voluntarily undertaken the preſent work with a view to inform our governors and every other claſs of men, of the experiments which others have made with the moſt happy reſult, as well as thoſe which I have myſelf conducted in conſiderable numbers, and with equal ſucceſs."

It may not be unacceptable to the Engliſh reader to have ſome extracts from Dr. Sacco's deſcription of the Cow-pox, as his ideas differ in ſome particulars from thoſe received in England, and as he aſſerts that the matter of the Cow-pox is to be found indigenous in Lombardy.

"The Cow-pox is a malady which, till the preſent time, entirely belonged to the veterinary department; and was ſo confounded with other cutaneous eruptions, that no one thought it worth particular attention. Nor is it to be wondered that this eruption was treated with ſuch negligence, for its mild nature and ſhort duration rendered its inconveniencies extremely trifling: Nay, there are ſome countries, where many aſſert, probably through want of attentive obſervation, that it does not exiſt in any ſhape whatever; and theſe people will have it, but without any foundation to their opinion, that this diſeaſe is excluſively confined to the weſt of England. From hence it proceeds, that ſcarcely a ſingle veterinary writer gives a proper deſcription and diagnoſis of it. There is another diſeaſe found among cattle, which reſembles the Cow-pox, but ought by no means to be confounded with it; as the former is a moſt contagious diſeaſe, and though not in itſelf of a mortal nature, it reduces the cattle to exceſſive leanneſs, and is in danger of deſtroying them if it be neglected at the commencement. The Cow-pox, on the other hand, is a diſeaſe equally contagious indeed, but ſo mild as ſcarcely to render the cow ſickly, yielding in a few days without any aſſiſtance, and effecting of itſelf a complete cure.

"Upon examining the diſeaſes which attack the cows of Lombardy, it appeared beyond a doubt that the Cow-pox is not peculiar to England; for upon examining our farmers, and thoſe who are uſually employed in the care of cows, we find that the herds, the cows of which are moſtly of the Swiſs breed, at certain times, particularly in the ſpring and autumn, are attacked with certain puſtules on their udders. Theſe puſtules, after paſſing through the ordinary ordinary ftages in the fame manner as the Small-pox, terminate in encruftations, which fometimes fall off in a fhort fpace, and at other times remain many days, occafioning a painful fenfation to the cows when they are milked, and thus incommoding thofe who are employed in that bufinefs. The fymptoms which ufually accompany this diftemper are, diminution of appetite, averfion to food, continual rumination without any materials in the mouth; and, to ufe the expreffion of the herdfmen who have obferved it, the cows make a motion with their lips, refembling that which men make when they play on the pipe. The milk then becomes lefs in quantity, and much thinner; the eye fomewhat downcaft. In the progrefs of the diftemper there appears a flight degree of fever, which is followed in two or three days by an eruption of fome puftules on the lower part of the udder, and particularly on the nipples. Sometimes, but rarely, a few appear about the eyelids and noftrils; thefe rife into little fwellings with their centre more depreffed, of a fhining appearance and a reddifh brown colour, and contain a thin inodorous fluid, which thickens, and remains in the puftules. Thefe, after two or three days, fuppurate, change into very thick encruftations of a deep red, and render it painful and difagreeable to the cows to be milked. Thefe encruftations, before arriving at their complete exficcation and detachment, pafs through a period of eight or ten days. This fpecies of diftemper, which is not very frequently obferved, is contagious to fuch a degree, that if one cow contracts it, in the courfe of ten days the whole herd is infected. There is another fpecies of diftemper exceedingly like this in the mode of infection and the progrefs of feveral of its fymptoms, but they differ in feveral particulars, the latter being accompanied with no fever, and being much fhorter in its duration, terminating in eight or ten days, during which period the encruftations, which are much fmaller than in the former diftemper, fall away.

" Such are the fymptoms obferved in Lombardy during the progrefs of Vaccine diftempers; and it is beyond doubt, that the Cow Pox found among the cows of Lombardy, is of a much milder nature than that known in England. This is evident from the defcription which I have given, and that given by Jenner of what he found in his own country.—I now proceed to give an account of the fymptoms which I have regularly obferved in thofe I have inoculated with the Cow-pox.

" Upon inoculating a man with the matter found in the puftules of the real Cow-pox above defcribed, feveral of its fymptoms refemble thofe obferved in the Small-pox; they are, however, diftinguifhed by one effential difference, which is, that the Cow-pox produces no general eruption, but remains limited to the arm, or to the very fpot where the matter was introduced. This difference alone is fufficient reafon for always preferring the Vaccine Inoculation; fince the Small-pox frequently produces an abundant eruption over every part of the body, which frequently becomes confluent, not only deforming the countenance, but even endangering the life. For the moft part, thofe inoculated with the Cow-pox are are attacked about the fixth or feventh day with a fever, ufually flight, but fometimes more fevere, and attended with cold and hot exacerbations, which laft one, two, or three days. This fever is accompanied with naufea, fometimes though rarely with vomiting, and fome convulfive movements of fmall moment. The fmall incifions or punctures made by the inoculation, at firft affume the appearance of a fly-bite, and at the fame time there is produced a flight degree of hardnefs in the cellular membrane, which is a certain fign that the inoculation is to take effect. Thefe punctures are afterwards furrounded with a red eryfipelous zone; they then fwell and rife into large puftules of a clear pale red, which retain a limpid humour till fuch time as they open of themfelves; at that period, which is ufually towards the feventh or eighth day, there proceeds from them a humour flightly vifcous, of a colour fcarcely tinctured with a very pale red, and by no means odorous; after fuppurating for two or three days, they become dry, and are converted into thick encruftations, which after eight or ten days more ufually fall off. It fometimes happens, particularly when the incifions are quickly converted into puftules and become foon exficcated, that a new inflammation fucceeds to the cruft already formed; the fever, which frequently is not perceptible during the firft period, now makes its appearance; fome pains are felt under the arm-pit, the glands fwell, the encruftations are furrounded with a rednefs brighter and more extended, new puftules of a large fize appear around the firft. Thefe puftules, containing the fluid above defcribed, after a new inflammation of three days, open of themfelves, fuppurate, and ufually become dry; fometimes but very rarely, the puftules are converted into little ulcers, which being fuperficial, are attended with no great inconvenience. Thefe laft very fingular phenomena ought never to be loft fight of by inoculators, that they may not run the rifque of looking upon the courfe of the Cow-pox as completed, or apprehend that the inoculation has mifcarried, when fome of its fymptoms may revive and crown the operation with the defired fuccefs."

The author afterwards quotes Dr. Jenner's defcription of the Cow-pox, and concludes that the fpecies found in Lombardy is of a milder nature than the Englifh. In treating of the origin of the Cow-pox, he does not admit Dr. Jenner's opinion about its being ultimately derived from the greafe of horfes. His obfervations on this fubject may be interefting to our readers.

" If we carried our account of the Cow-pox back to the origin of contagious diforders and their true caufes, we fhould involve ourfelves in a maze of obfcurity, and contribute nothing to the advancement of fcience. According to the affertion of Jenner, the origin of the Cow-pox is to be deduced from another diftemper not proper to this animal. He alledges, that the exciting caufe of this infection arofe from another difeafe to which horfes are fubject, called among the Englifh the greafe, and that thofe people who were employed in the cure of fuch ulcers, going afterwards to milk the cows with this matter on their hands, communicated municated to them a new fort of diftemper. Thefe affertions are nothing more than fimple conjectures; but as they come from Jenner, fupported by various facts, they may on this account feem worthy of notice and difcuffion. Nay, more, the Englifh writer will have it, that not only the matter which iffues from the *grease*, but matter from any other equinous ulcer whatfoever is able to produce in cows the fame infection. I fubjoin fome obfervations on which Jenner has founded his theory and refts his opinions." After quoting fome facts adduced by Dr. Jenner in fupport of his theory, Dr. Sacco proceeds: " From thefe facts Jenner concludes, that the introduction of the *greafe* into the human fyftem, may, perhaps, preferve it from the attacks of the natural Small-pox; but as in many cafes the refult of his obfervations is not conftantly the fame, the Small-pox fometimes producing an equivocal eruption, he thinks it neceffary that the pus of the greafe, or any other equine ulcer fhould come newly modified through the medium of cows. This much is certain, that if the origin of the Cow-pox can and ought to be attributed to the greafe, as the genius of the diftemper is different in horfes and cows, it appears that the pus, on being transfufed into the latter, undergoes a confiderable modification, and becomes in fome meafure effentially changed. The facts adduced by Jenner appear to give fufficient weight to his conjectures, and to juftify the conclufion that the Cow-pox may draw its origin from the ulcers of horfes. The following obfervations may, neverthelefs, throw fome dubiety on thefe conjectures. Jenner's explanation of the fact is plaufible enough with regard to the province of Gloucefter, where the fame perfons are entrufted with the care of both horfes and cows; but in Lombardy, on the large farms, where the herds of cattle are very numerous, the departments of the attendants are divided, the cows are committed to the charge of one clafs of perfons, and the horfes to that of another; and thefe two claffes do nothing in common, nor is the one ever employed to difcharge the offices of the other. Ireland, fays Jenner, which abounds fo much in cattle, is, till this hour, free from the Cow-pox; and the reafon he gives for this exemption is, that the cows are milked only by women, and that a man would reckon himfelf difhonoured were he employed in this office. The combination of thefe circumftances among them is in oppofition to what is obferved in Lombardy, where, notwithftanding that different perfons are employed about the cows and horfes, the former are fubject to this diftemper. There can, therefore, no ftrefs be laid on the confequences which Jenner would deduce, and his theory can only be regarded as an hypothefis not properly fubftantiated. The origin of the Cow-pox then muft neceffarily be fought from fome other fource; and if, after all, neither obfervation nor reafoning be able to difcover it, it muft remain in the fame obfcurity with many other fubjects of difquifition which have fruitlefsly and without any utility occupied the attention of theoretical phyficians and other learned men. Facts, alone, ought to be received as a confirmation of of the conjectures which propofe to deduce the origin of the Cow-pox from equine ulcers; and thefe facts ought to be univerfal and conftant. The only method to confirm or overturn fuch theories is, to inoculate cows with the pus of the greafe, in the fame manner as men are inoculated with the Cow-pox. A repetition of the experiments will fhew whether it be contagious, and whether it really paffes from the horfes to the cows, or whether the latter come to be attacked with it in confequence of fome other unknown combination. Jenner, as far as I know, has made only one experiment, and has not obtained the refult, which his theory feems to require, of producing the Cow-pox. In order, then, completely to confirm the theory of Jenner, or directly to confute it, I undertook to make divers experiments on a certain number of cows; four were the number fet apart for this attempt. I began with making fome incifions with a lancet in the udder and nipples of one; I then introduced the frefh matter of the greafe, which an expert groom, of Milan, had purpofely procured me that very morning. As the animal was very reftlefs during the time of the experiment, I deferred till next day the inoculation of the other three, wifhing to avail myfelf of an inftrument ufed in Germany by phlebotomifts, as by means of it I could render the transfufion of the matter of the greafe into the cows more frequent, more eafy, and more decifive, on account of the multiplied apertures. Having charged the inftrument with fix lancets, I applied it at the roots of each pap, and by this means produced in all twenty-four fmall incifions, which became gently tinged with blood. Not only the place where the incifions had been made were filled with the greafe matter, but the whole paps were covered with it. I inoculated the other two in the fame manner with the greateft facility. I flattered myfelf that by this method the abforption of the pus could not fail to take place and to produce the defired effect, as it was rendered powerful both by the number of the incifions and the quantity of matter. Having covered the wounds where the pus had been introduced, with a piece of linen, around which pitch was laid, fo that the matter might not be expofed to the air, or in any way removed from the place; contrary to my expectation, there was no fenfible appearance on any of the cows, and the incifions dried up in two or three days, without producing any alteration whatever. Perhaps, the non-appearance of the effect I looked for may be attributed to the feafon of the year, as the autumn, in which I made the experiments, is not the moft favourable to exanthematous eruptions, the contrary of which is obferved of the fpring. Perhaps there was wanting a predifpofition to contract the diftemper; or, perhaps, there was wanting fome circumftances, unknown to us, but neceffary to make it unfold itfelf. Perhaps, and it is a more probable fuppofition, the effect was not attained, becaufe the greafe is not the origin of the Cow-pox. I ingenuoufly bring forward my refults, although I do not place implicit confidence in them, but prepare myfelf to repeat new experiments at different feafons of the year, which other medical men have alfo alſo an opportunity of doing, in order to be convinced, whether they ought to receive or reject the conjectures of Jenner. Let me add to the cauſes aſſigned to the Cow-pox, the opinion of thoſe who deduce it from a different origin, and regard it as originating in the cows themſelves, and proceeding from a particular ſtate in which they are at times found. It has been obſerved, that the diſtemper uſually appears in cows which have newly calved: from thence it is ſuppoſed that the milk, not finding the proper veſſels ſuddenly prepared for its diſcharge, becomes acrid, irritates the dugs, occaſions inflammation, and ſo produces this diſtemper. And, beyond doubt, when the milk is retained in the udder beyond the period aſſigned by nature, it contracts ſtimulating qualities, from which they may ſuffer; I do not however believe, that this circumſtance can give riſe to the Cow-pox, which is a contagious diſtemper. The theory is nowiſe ſupported by obſervation, as cows in the ſtate here deſcribed are to be found in every place. Another argument againſt this hypotheſis may be drawn from the analogy of thoſe ladies who follow the barbarous faſhion of not ſuckling their own children, and who of courſe retain the milk in their breaſts even till it acquires a great degree of acrimony. Although in ſome of them there have been obſerved exanthematous eruptions producing thick incruſtations and ſevere pains, there never aroſe from theſe ſymptoms, in one inſtance, a diſtemper of an infectious kind. I will not leave this ſubject without giving my own ideas, founded on many obſervations, and already advanced by many other medical men on the origin of exanthematous diſeaſes, among which both the Small-pox and Cow-pox muſt be comprehended. This theory ſuppoſes that ſuch diſeaſes are produced, or at leaſt always accompanied, by worms, which inſinuate themſelves into the ſkin, and afterwards neſtle in the puſtules, which grow up and come to a ſort of maturity. Etmuller and Hoffman, among the older writers, are of that opinion with regard to many diſeaſes; and ſome of the moderns, founding their ideas on incontrovertible microſcopic obſervations, look upon the itch and other contagious diſeaſes as originating from worms. Why may not the variola be of the ſame nature? I have hitherto had no opportunity of making thoſe reſearches on this ſubject which I have of a long time reſolved to engage in; as I have not been able to procure microſcopes of ſuch power as to enſure certain reſults. It is well known that an excellent method of cure in diſtempers from worms is the uſe of mercurial medicines, which have alſo been employed with ſucceſs in the itch. When applied to the variola it ought to have the ſame effect, provided this fatal and contagious diſtemper can be attributed to worms which are lodged in its puſtules. The celebrated Roman phyſician, Dr. Lapi, has publiſhed in an excellent work, his ſucceſsful experiments on the uſe of mercurial ointments in the variola, without however mentioning to what cauſe he attributed this effect, and without generalizing his theory and extending it to other exanthematous diſeaſes which are not contagious; to theſe it would have been proper in him to have applied the ſame treatment. I have myſelf repeated ſimilar experiments on the variola in a few caſes, cases, and the result was conformable to that obtained by Doctor Lapi."

Before proceeding to state the cases of Vaccine Inoculation which occurred in his own practice, Dr. Sacco gives the following account of the manner in which he procured the pus for inoculation. "I had for some time been extremely desirous to repeat the experiments of Jenner, and for this purpose made diligent researches to discover the Cow-pox in Lombardy, it being extremely difficult, especially in the present circumstances, to obtain the pus from England. A fortunate combination of circumstances, by which it became necessary for me to go to the large town of Varese, in the beginning of autumn, procured me an opportunity of examining a number of cows on their way from Switzerland to the fair of Lugano; and by this means I had a favourable opportunity to make such researches, as might discover in some one of them the Cow-pox. It was on this ocasion, that conversing with some dealers in cattle, and countrymen who had large dairies in lower Lombardy, I learnt that the cows among us are subject to the Cow-pox. In this enquiry I took care to propose my questions in such a manner, as to prevent the risk of being imposed upon. A farmer of Cremona, who had bought forty cows in Switzerland, and had driven them from thence as far as Varese, assured me that almost all of them had been successively attacked with pustules on the extremity of their nipples, and some of these were now converted into incrustations. I visited the cows, and had an opportunity of verifying his assertions. I picked off some of these encrustations, with an intention of applying them in fomentation, if, perchance, I could not procure the true pus for inoculation. The same farmer promised me an opportunity of seeing this disease with my own eyes, and for this purpose conducted me to a neighbouring meadow, in which we found a herd of cows belonging to a friend of his. We examined these cows, and discovered on two of them different red spots, which the farmer assured me was the first stage of the disease; no other symptom appeared on the cows, but a slight degree of dejection. He assured me that this was the very disease I was in quest of, and that in the course of two days, the pustules would unfold themselves. At this visit which I made to the cows, there was present a dealer in the Grisons cows, who fully confirmed the truth of these assertions. He also added, that in his country he had seen the cows afflicted with a similar eruption on their dugs, and to remove the incrustations it was common to anoint them with boiled oil used for varnish; and that by this means they fell off in the course of two or three days. Early next morning I went again to see the cows, examined them anew, and found on one of them four red spots already tumid and raised into pustules; three of these were spread over the nipples, and the fourth lay in the middle of the dugs. The other cow had six pustules, two on the nipples, and the rest scattered above them. These were larger than those of the first cow, and around them appeared a slight flight red circle. Apparently thefe puftules occafioned much pain to the cows, for on my approaching to examine them more minutely, they would fcarcely permit me to touch them for one moment. Although the puftules were already large and prominent, they did not yet appear to me fufficiently mature to yield the matter I wanted. As the cows were that day to go forward on their way to Milan, I found myfelf under the neceffity of following them to their firft halting place, in order to examine them again next day. I walked out at an early hour to the meadow where they were at pafture. I examined the puftules, which appeared to me to be now arrived at maturity. They were lucid, and of a pale red colour, with a brown fpot in the middle more depreffed, and I thought this a favourable moment to collect the matter, which, through the affiftance of the herdfmen, I was eafily enabled to do by repeatedly foaking a thread in it. Although I faw no reafon to doubt that this was the true Cow-pox, yet this being the firft time I had ever feen it, I began to fufpect that the puftules might be of that kind which Jenner calls the *fpurious* Cow-pox; I determined therefore to decide the matter by experiment. A confiderable number of experiments, all uniform in their fymptoms and progrefs, and always conftant in their refults, put the matter beyond doubt, and gave me full conviction that this was the true Cow-pox. Such and fo many are the obftacles to be overcome on the introduction of any innovation, however falutary, that I for fome time defpaired of being able to induce any one to fubmit to inoculation with the matter I had collected. In fine, after many fruitlefs perfuafions, I fucceeded in my defign; the fuccefs which attended the firft inoculations encouraged others to fubmit to the fame procefs."

Dr. Sacco then proceeds to detail three hundred cafes, in which he applied the pus he had obtained in the manner defcribed above. Thefe cafes were attended with various circumftances; but the inoculation fucceeded to produce the Cow-pox in all of them; and in a confiderable number the inoculation for the Small-pox was afterwards applied, but without any effect. Having learned from a veterinary furgeon in the department of the Eure and Loire, that not only oxen, fheep, and rabbits, but dogs alfo were fubject to the effects of the Vaccine inoculation, Dr. Sacco extended his refearches likewife to this fubject, reckoning no part of phyfical knowledge a fruitlefs or barren difquifition. He ftates the refults he obtained as follows. "I took feven dogs, of different fpecies, and fubjected them fucceffively to the Vaccine inoculation, conftantly employing the matter obtained from the preceding to inoculate the one I next intended to fubmit to the operation. The difeafe was produced on them, but had a much fhorter period than it ufually has in the human fpecies. Two of the dogs were for the fpace of one day extremely dejected, and refufed all manner of food; but during the reft of the period they appeared perfectly well, nor was there any other confequence that merits obfervation, except that the Vaccine Inoculation tranfmitted from one dog to another preferved always the fame force, and never loft its activity.

"In the firft five of thefe experiments I applied that for the Small-pox after the Vaccine Inoculation; I had not an opportunity of trying the fame operation on the fixth, as he had to attend his mafter on a journey. On the feventh, fome time after the incruftations had begun to fall off, fufpicious fymptoms of the hydrophobia began to make their appearance; his habitudes underwent a rapid change, he became unwilling to drink, bit fome perfons, and in confequence was put to death. None of the perfons he had bitten were attacked with hydrophobia. Such were the confequences of the inoculation of the Small-pox practifed on dogs after the Vaccine. I afterwards inoculated the Small-pox on two dogs who had not been previoufly vaccinated; and one of them had three puftules on his lips, and one in the vicinity of the inoculation; on the other, one only appeared in the neighbourhood of the inoculation. During the courfe of the difeafe, thefe dogs were for fome days melancholic, and ate much lefs than ufual. From thefe experiments it appears, that the Vaccine Inoculation acts as a prefervative in dogs againft the Small-pox. The fymptoms which I obferved in my Vaccine inoculation of dogs do not enable me to form a precife judgement, whether it is attended with any inflammation, great or fmall, on the lungs; fo that I am unable either to oppofe or coincide with the fentiments of the illuftrious Englifh phyfician on this fubject."

The Doctor then proceeds to give an account of the method of inoculation, and the treatment of the Cow-pox, but adds no new obfervations to the compleat information already poffeffed in England on thefe fubjects. An extract from the confequences and reflections he has deduced from his experiments and obfervations will probably be acceptable to the Englifh reader.

"1ft. The Cow-pox appears to be a diftemper incident to every fpecies of that animal, fince it is not confined to England, but is alfo known in Holftein, is extended to Switzerland and Lombardy, and we may conclude, without hefitation, that it is alfo to be found in other countries. The caufe that has brought this circumftance into doubt, is the ignorance of its merits, and the confequent inattention of intelligent perfons. In fact, no one had the leaft knowledge of it in Lombardy, nor would even have fufpected its exiftence there, if the refearches which I undertook, following the footfteps of Jenner, had not fucceeded in difcovering it. I have now ufed that matter, which I found out in the manner above defcribed, in fuch inoculations as I have been engaged in during the laft eight months. Other medical men of Lombardy alfo, though they have not difcovered precifely the fame fpecies of variola, neverthelefs entertain the beft founded fufpicions that it exifts among our cows; and from the replies which I have received to my enquiries on that fubject, I am inclined to believe, that if attentive obfervers would employ themfelves in this refearch, the common and general exiftence of this difeafe among our cows would foon be be eftablifhed. Moreover, the Cow-pox might be introduced and propagated without the neceffity of bringing the pus from diftant countries, as is the practice throughout all Europe, to every country of which it is brought from England with a fuccefs not always to be relied upon, fince it lofes its activity by the length of time that muft neceffarily intervene before it can be applied to ufe, and is thus in danger, in place of the true Cow-pox, of communicating the fpurious, which does not preferve from the attacks of the Small-pox.

"2d. It appears that the vaccine variola ought to be regarded as a modification of the human, and if they differ in fome degree with regard to the feat of the malady and the method of its communication, they agree in the remainder of their effential qualities. The human variola is communicated from one fubject to another by the means of miafmata invifibly tranfported through the air; it is communicated by the mere contact of found perfons with the infected, and even by handling the clothes of thofe who have the difeafe. The vaccine variola, on the contrary, is only communicated by means of infertion; by directly bringing the matter of it into contact with the mafs of the blood by means of flight incifions or punctures, or by ftrong and long-continued friction. This laft is the means by which it is communicated to thofe who are employed in milking the cows; the operation warms and opens the pores of their hands, by which means the pus that is fqueezed by compreffion out of the puftules has an opportunity of introducing itfelf through the abforbent veffels at the extremity of the capillary veins. The human variola difcovers itfelf on every external part of the body without exception, and even on the internal, as the mouth and jaws; while the vaccine may be confidered as purely local, that is to fay, limited to the margin of the incifions, or punctures, through which it was introduced; and there alone it produces a hardnefs, an inflammation, and puftules. The diftance of time between the inoculation and the appearance of the difeafe is not conftant, either in the human or vaccine variola. In both, the firft fymptoms begin to appear about the fifth day, or the days immediately fucceeding; fometimes not till the thirteenth, the twentieth, and even the fortieth day.

"3d. It appears a rafh affertion of Jenner, that the greafe of horfes is the exciting caufe of that difeafe in cows which is called the Cow-pox. I have proved the falfity of this opinion by direct experiments and obfervations; and I have fince been informed that Pearfon, Simmons, and Woodville likewife entertain fentiments on this fubject oppofite to thofe of Jenner.

"Jenner wifhed to extend the effects of this matter fo far as even to confider it as the general caufe of all contagious difeafes; and in order to render the conjecture in fome degree plaufible, he believes that this matter, upon being modified and changed in the human body by certain compound and unknown means, may afterwards unfold its activity in fome Protean form. But as it has been demonftrated that the fimple matter of the greafe, which is regarded regarded by Jenner as the exciting caufe of the Cow-pox without any modification, is not fo in any way whatfoever; it ought with ftill more reafon to be excluded from being the origin of other contagious diforders; a theory without the fmalleft proof or foundation.

4th. From numberlefs experiments, it may be looked upon as a demonftrated maxim, that whoever has had the Cow-pox is no longer fubject to the attacks of the Small-pox.

" 5th. I look upon it as alfo demonftrated, that the true Cow-pox cannot be produced in thofe who have had the natural Small-pox. I have above detailed different experiments which I have made with a view to this queftion: I will now add that the celebrated Dr. Arragoni, who had in his early years the confluent Small-pox, made me repeat on him the Vaccine inoculation with frefh matter, and with all the precautions neceffary to produce the true Cow-pox; but the only refults obtained were thofe of the fpurious. It is a general received opinion in England, as different Englifh Inoculators have advanced in their works, that thofe who once had the natural Small-pox are no longer liable to the Cow-pox. I know that Jenner and Pearfon are of a contrary opinion. Few are the facts on which they reft their theory, and thofe few are only fuch as Jenner has produced in his firft work. My own experience, neverthelefs, and the many obfervations I have made, confirm me in my own opinion, and furnifh me with a criterion by which to determine whether thofe, who doubt if they have had the natural Small-pox, have or have not been attacked with it.

" 6th. Where the proper effect has been produced on thofe vaccinated, they are no longer capable of contracting the difeafe a fecond time. This conclufion is in oppofition to the opinion of Jenner and Pearfon; but to their authority I oppofe the refults of my own experiments, and thofe of other inoculators. Befides the different attempts which I made to vaccinate different fubjects a fecond time, I alfo fubjected myfelf to the fame experiment: but the only refult I obtained, alike from them and myfelf, was the fpurious Cow-pox, of a fhort period, and producing in fome of them a flight fever. In this particular the human variola agrees with the vaccine, as both of them, on a fecond inoculation, produce only tranfitory fymptoms.

" 7th. From above two thoufand vaccinated perfons having lived with thofe who were not vaccinated, yet without communicating the difeafe to the latter, we may conclude that it is not contagious in the human fpecies. Woodville alone holds a contrary opinion; but as he adduces only two facts in oppofition to fo many, it is probable he has been mifled by an anomalous appearance of the natural Small-pox during the courfe of the Vaccine inoculation.

" The true Cow-pox is by no means to be confounded with that which has been denominated the *fpurious*. It would be a fatal error to employ the one as equal in effect to the other. The *true* preferves from the Small-pox, the *fpurious* does not. Thefe two difeafes have their diftinctive characters; and I will not fcruple to repeat, repeat, that it is of the utmoſt importance to be able to diſtinguiſh them at firſt ſight; this alone can form that tactus eruditus which is the reſult of repeated experiments, of ſome errors, and of accurate obſervations. It will not therefore be ſuperfluous to repeat the obſervations I have made on this article. The true Cow-pox does not ſhew itſelf before the 4th day; ſometimes not till the 7th, the 8th, or even later. Its firſt ſymptom is the appearance at the place where the punctures have been made, of a ſpot like that which is occaſioned by a flea-bite, with a very minute elevation towards its centre, as may be ſeen in Figure 2, Plate II. Two or three days after the appearance of the afore-mentioned redneſs, this elevation augments in diameter, and takes the form of a ſmall puſtule or tumour. The red ſpot increaſes in proportion, and now forms a ring of larger circumference, as may be ſeen in Figure 3. About the 5th or 6th day it arrives at its completion. It is of a circular figure, and its diameter is from three to five lines; it has elevated borders of a clear aſh colour; its centre, which is of a reddiſh brown, is depreſſed and filled with a limpid cryſtalline matter. It now becomes ſurrounded with a red ring of a larger diameter than that of the preceding days, (as may be ſeen in Figure 4:) this ring continues to expand itſelf with increaſing rapidity, acquiring a magnitude very well repreſented by Fig. 5, and embanked as it were by a ſlight miliary eruption. At the ſame time the red colour undergoes various degradations, being always moſt intenſe in the moſt eccentric parts. Such parts as are reddiſh at this period, eſpecially in the vicinity of the puſtule, contract a hardneſs in the cellular membrane ſimilar to what takes place in an eriſypelas. An incruſtation commonly ſucceeds in place of the puſtule, without any ſuppuration; and at the ſame time the eriſypelous tumour begins by degrees to wear off; the incruſtation preſerves in every part the exact figure of the puſtule, and preſents a ſurface, bright, ſmooth, and as it were pellucid, of which ſome idea may be formed from Fig. 6. Towards the third or fourth day of the appearance of theſe ſymptoms, the perſons vaccinated are attacked by ſome degree of fever, accompanied by debility and want of appetite. The duration of the febrile ſtate is uſually confined to a few hours; in ſome perſons it returns a ſecond time, but very ſeldom is again renewed. At the ſame time that the fever makes its appearance, there begin to be felt pains under the arm-pits, attended with a tenſion of the glands, which ſymptoms regularly diſappear when the fever goes off. It ſometimes happens, that after the formation of the puſtule is completed, it opens and ſuppurates. In that caſe the incruſtation by which it is covered has an unequal ſurface, an unequal colour, variegated with yellow and red, and is likewiſe of a large ſize, as may be learnt from Fig. 7. The duration of both theſe incruſtations is about eight, ten, and ſometimes fifteen days before they become diſengaged from the ſkin. They ſometimes fall off, and are re-produced again and again, always occaſioning ſome ſuppuration. This deſcription comprehends a caſe of the Cow pox accompanied with well-formed puſtules, and which therefore cannot be be miftaken. It frequently happens, that fuddenly, and in the courfe of a few hours after the inoculation has taken place, efpecially if it has been performed by means of threads, or with matter which has been long kept, the firft fymptoms begin to appear, inflammation at the feat of the punćture, the rapid formation of a puftule containing matter, and moft commonly alfo a fenfible fever accompanied with vomiting. The puftule opens of itfelf, and in the fpace of about fix days after the operation has been performed, the exficcation fucceeds; no veftige of the puftule then remains except a flight incruftation, which often continues to ooze out matter for a long time afterwards. Such are the fymptoms that excite fufpicion of their being derived from the fpurious Cow-pox. I have applied myfelf with that folicitude which the importance and difficulty of the fubjećt required to form a precife diagnofis, which may render us fecure of not miftaking the fpurious for the true.

" If after the above-mentioned fhort period, the difeafe entirely vanifhes, it is certain that the fymptoms which have appeared are thofe of the fpurious. On the contrary, if, after that fhort period, the inflammation takes place anew around the encruftations; if, in place of them, an elevation and fwelling are formed in the cellular membrane, with an external erifypelous appearance; if on the falling off of the encruftations the wound opens afrefh and begins to fuppurate, and has nearly every fymptom that ufually accompanies the true Cow-pox; we may conclude that the true has been obtained, its fymptoms having fucceeded to the former dubious ones. The fpurious Cow-pox is fuch as has been defcribed above. The moft effential of its indications is the great celerity with which it appears after the innoculation has taken place, while the fymptoms of the true do not make their appearance till after four, five, or more days. The encruftations of the fpurious are fmall and irregular; while thofe of the true are much larger, of a regular figure, and relucent. It will be afked, What is the origin of the Spurious Cow-pox? From what Jenner fays, and from what I have myfelf obferved, it takes place whenever the matter for inoculation is extraćted from puftules originally fpurious, fuch as I have already noticed in my defcription of the Cow-pox: It takes place when the matter is from puftules already approaching to exficcation, and in a ftate of fuppuration: It takes place when the virus ufed for inoculation has been long kept, and altered by heat or any other caufe, and therefore little reliance is to be placed in that tranfmitted from diftant countries, unlefs it arrive quickly at the place of its deftination: It takes place when the matter of inoculation is taken from that already known to be fpurious from its courfe: It takes place whenever the true kind is ufed in the inoculation of any one who has already had the natural fmall-pox, or who has already been vaccinated. Thofe who wifh to know the reafon of every thing, will expećt to learn from me, why, in thefe laft cafes, inoculation with the matter of the true Cow-pox produces only fpurious, by which means a criterion is formed formed to judge whether Vaccination or the natural Small-pox has already taken place. I confefs my ignorance in common with all thofe who have already publifhed the fame obfervations, and we muft ftill wait an explanation of the phenomenon from time, from fome fortunate accident, or from new obfervations that may rend afunder the veil of this medical myftery.

Dr. Sacco fubjoins to this work a plan to render the ufe and the advantages of the Cow-pox general. The methods which he points out for this purpofe are, 1ft. To form a public inftitution for inoculating gratis, fituated in the capital, and communicating with fimilar fubordinate inftitutions to be eftablifhed through all parts of the kingdom. The principal inftitution to have the charge of fupplying any deficiences of the matter of inoculation, and alfo of circulating any new improvements or farther difcoveries of the diagnofis and treatment. 2d. All admitted into the Foundling, Orphan, &c. and all other public hofpitals and inftitutions, to be vaccinated immediately on their admittance, or on one day of every week fet apart for that purpofe. 3d. No medical man fhall be admitted to any employment in the public fervice, without firft entering into an obligation, on pain of forfeiture of his office, to vaccinate all thofe *gratis* who fhall apply to him for that benefit. Such are the leading principles of Dr. Sacco's plan: the detail of it is adapted entirely to his own city of Milan and the furrounding country, and therefore could yield no information of general utility. He concludes with a hope, that the adoption of this plan would fpeedily introduce a cuftom of vaccinating all new-born children, and thus in a fhort time deliver the world from that dreadful malady the natural Small-pox.

☞ *The interesting Plate representing the Cow-Pox as it appears in Lombardy on the Udder of a Cow, was not ready in time, owing to the neglect of the Engraver, but it will certainly appear in the next Number.*

---

*Observations et Experiences sur l'Inoculation de la Vaccine, par* JEAN DE CARRO, Docteur en Medecine. 8vo. pp. 216. Vienna, 1801.

THE Author of this excellent work on the new Inoculation may be confidered as the principal founder and promoter of it in Germany, Ruffia, and Turkey. In the Dedication to Lord Minto, our Ambaffador at Vienna, he fays, " The healing art is indebted to England for a multitude of ufeful difcoveries; no epoch, however, has been fo memorable, nor has promifed to mankind advantages fo important as Vaccine Inoculation, the immediate tendency of which is to annihilate that deftructive fcourge the Small-pox.

" England already acknowledges Jenner to be one of her greateft benefactors, and all civilized nations haften eagerly to profit by his admirable difcovery. Having had the good fortune to be the inftrument of its introduction into the Auftrian monarchy, I owe

 to to the reprefentative of Great Britain the homage of my firft fuccefs."

In his Preface he announces the end propofed by the publication of this work to be, "To collect the proofs of the prefervative power of Vaccine Inoculation, to lay them before the public in a methodical and accurate form, to examine the opinions of the moft celebrated inoculators on all the interefting points relating to the Theory and Practice of Vaccination, and not to give my own opinion, fays Dr. C. till after the refult of numerous obfervations and experiments which I have had occafion to make upon this new inoculation.

"In order to facilitate as far as poffible the circulation of a work, written for parents as well as medical practitioners, I have had it tranflated into German, under my own infpection, by Dr. De Portenfchlag, junior."

Our readers will judge of the *extent* of this elaborate work, by the following heads of the feveral chapters.

1. Remarks on the name of the difeafe. 2. Hiftory of the difcovery of the Vaccine Inoculation. 3. Origin of the Vaccine. 4. Defcription of the true appearance of the vaccine puftule, with a plate. 5. On the poffibility of having the difeafe repeatedly, and the Vaccine after the Small-pox. 6. Can it be communicated by any other means than inoculation? 7. Obfervations on the eruptions which have been obferved in the Inoculation Hofpital at London, and fome other places. 8. Is it difficult to propagate the difeafe, and keep up a fupply of the virus out of England? 9. An account of the Inftitution for the Inoculation of the Vaccine in England, and rereflections on the neceffity of fuch eftablifhments. 10. Is it neceffary that there fhould be a decided fever to be affured of the anti-variolous power of the Vaccine? 11. Are the Vaccine and the Small-pox the fame difeafe differently modified? 12. Of the immediate and remote advantages of the Vaccine over the Small-pox. 13. Are there any well-founded objections to the adoption of the Vaccine? 14. Are there any authentic cafes where the Small-pox has appeared after the Vaccine? 15. Obfervations on the practice of the new inoculation. 16. A lift of my inoculated patients.

To which is added an appendix of fixteen pages, containing letters from his correfpondents.

In his fecond chapter on the hiftory, he fays, "I ought not to forget the correfpondence with my Lord Elgin, Ambaffador Extraordinary of his Britannic Majefty, at Conftantinople. I have had, through his means, the pleafure of contributing to pay a part of the debt we owe the Turks for teaching us the inoculation of the Small-pox. My Lord Elgin, convinced of the importance of Dr. Jenner's difcovery, and the authenticity of the facts upon which it was founded, applied to me for virus, to inoculate his only fon Lord Bruce, in which he fucceeded compleatly." Then follows a copy of Lord Elgin's letter to Dr. De Carro, on which he remarks. "Here we fee an Ambaffador from England, acting the fame part with the Vaccine in Turkey, as an Ambaffadrefs (Lady W. Montague) had acted in Europe on her return from Conftantinople.

"Laftly,

"Laftly, Of all the proofs which I could give of the high importance which the Englifh nation attaches to the difcovery of Dr. Jenner, none appear to me more decifive than the order given by the Admiralty for the inoculation of all the children of failors, and the failors themfelves who had not undergone the Small-pox; not only in the ports of Great Britain, but in the moft diftant fettlements. When we reflect for an inftant, that this clafs of men conftitutes the power and the glory of that Nation, can we doubt the advantages, or the authenticity of this inoculation?"

Dr. De Carro's plate reprefents the puftule fomething fmaller than it appears in London; and we are informed that it is fmaller in England, in the country, than in London.

In his chapter on the eruptions, he fupports the opinion that is now, we believe, generally received here, viz. that they have nothing to do with Vaccination. He concludes it thus, "I am at prefent fo convinced of the fafety of the Vaccine, that I pay no attention to the ftate of the child's health before inoculation. The weakeft as well as the ftrongeft go through it with the fame eafe. In a word, I repeat here what I have fo often faid to apprehenfive parents, *If a child is not dying of some other disease, he will not die of Vaccine.*"

Near the end of his eighth chapter, on the means of continuing the true virus, "How many country practitioners, who were not fufficiently acquainted with the difeafe, rafhly inoculated with what they confidered as the true Vaccine, but which was fpurious. So that I ftrongly recommend all perfons who value this difcovery, to diftruft all unfavourable reports made by people who do not give the genealogy of their virus. This precaution is the more neceffary, becaufe I certainly know that fome interefted enemies of the new method, have feveral times tried with virus, which they inferted as vaccine, but which was fpoiled by heat, or defignedly mixed with variolous matter, in order to produce confequences which might create a general difguft againft Vaccine Inoculation."

On the queftion refpecting the neceffity of a conftitutional affection or fever as forming a part of the genuine difeafe, Dr. De Carro declares, "that whenever the vaccine puftule, or rather vefìcle, affumes its true character, which he fays may be afcertained by the plates of Dr. Jenner, or thofe in the prefent work, we may fafely conclude that the patient will be for ever fafe from the Small-pox."

We cannot help regretting on this occafion that Dr. Jenner's engagements prevent him from giving to the public thofe very accurate and beautifully coloured plates which he is now preparing to accompany the next edition of his works on Vaccination. Thofe plates would indeed be a rudder and a compafs by which the practitioner might fteer with fafety.

"After the opinion I have juft given, (fays Dr. De Carro) how can it be conceived that an effect apparently merely local, can guard againft fuch a difeafe as the Small-pox, whofe effects on the general fyftem are fo violent and fo well known? Certainly, the

 fact fact is very extraordinary; it is a new myftery added to thofe which, from the commencement of medical fcience, have been deplored by its Profeffors.

" Few people, it appears to me, can fuppofe that the variolous action is merely local, fince fuch numerous experiments in various climates prove the contrary. I believe that a conftitutional affection does always exift, but that it is fo flight as often to efcape the notice of the inoculated and the Inoculator. According to my obfervation, in thofe families where the children have been watched with the greateft attention and care, the moft conftant fymptom has been reftleffnefs during the night of the 9th and 10th day. It is obvious, that fymptoms fo flight as thefe will efcape the notice of the generality of parents, and more fo that of phyficians, who are fo feldom with their patients at thofe hours. But though no fymptom of fever has been noticed, we cannot conclude that it had no exiftence. Fortunately for the fuccefs of this inoculation, we certainly know, that the appearance of the areola, is a decided proof that the anti-variolous change has been produced in the fyftem; and independent of this circumftance I know no means by which the practitioner can be affured that the true difeafe has taken place.

In the eleventh chapter he draws this conclufion. " Dr. Turton is the only one who has concluded that Variola and Vacciola are the fame difeafe differently modified; which opinion, I think, is fufficiently refuted by what I have advanced above. It is more reafonable to fuppofe that they are diftinct difeafes, the one originating in the cow, the other in man; but that they have a certain relation which cannot be defcribed nor explained. The difcovery is a new and unique fact in Phyfiology, Pathology, Natural Hiftory, and Veterinary Science."

Dr. De Carro concludes his next Chapter in thefe terms. " In the courfe of the laft autumn and winter, five cafes of Inoculation occurred in children who had before received the infection of the natural Small-pox, and in whom that difeafe appeared a few days after the Vaccine Inoculation. Thefe five children had the Small-pox aftonifhingly mild, at a time when that epidemic was uncommonly fevere. The two maladies proceeded together with the greateft regularity, and each preferved its peculiar character without approaching the other."

